# Frailty index is an independent predictor of all-cause and cardiovascular mortality in Eastern Europe: a multicentre cohort study

**DOI:** 10.1136/jech-2023-221761

**Published:** 2024-08-24

**Authors:** Tatyana Court, Nadezda Capkova, Andrzej Pająk, Abdonas Tamosiunas, Martin Bobák, Hynek Pikhart

**Affiliations:** 1 RECETOX, Faculty of Science, Masaryk University, Brno, Czech Republic; 2 National Institute of Public Health, Prague, Czech Republic; 3 Department of Epidemiology and Population Sciences, Institute of Public Health, Jagiellonian University Medical College, Krakow, Poland; 4 Laboratory of Population Research, Institute of Cardiology, Lithuanian University of Health Sciences, Kaunas, Lithuania; 5 Research Department of Epidemiology and Public Health, University College London, London, UK

**Keywords:** AGING, MORTALITY, COHORT STUDIES

## Abstract

**Background:**

This study investigates the association between frailty and mortality in Eastern European populations, which remains largely unexplored compared with Western Europe. The aim is to assess the risk of all-cause and cardiovascular mortality associated with varying levels of frailty.

**Methods:**

A prospective multicentre cohort study was conducted, involving random population samples from the Czech Republic, Poland and Lithuania. The baseline survey (2002–2005) included 26 746 individuals aged 45–69 years, with an average follow-up of 13 years. Frailty was measured using a Comprehensive Geriatric Assessment (CGA)-based Frailty Index (FI), calculating the number of deficits in each domain. Cox proportional regression models and inverse probability weighting (IPW) were employed to account for risk factor differences among the frailty groups: robust, prefrail, mild, moderate and severe.

**Results:**

The study included 14 287 people, among whom 891 were frail, with a total of 2402 deaths.

Compared with non-frail persons, those with mild (IPW HR 2.06, 95% CI 1.60 to 2.66) and severe (IPW HR 2.71, 95% CI 1.45 to 5.07) frailty had more than twofold elevated risk of all-cause mortality. For cardiovascular mortality, the corresponding HRs were (IPW HR 3.05, 95% CI 2.14 to 4.35) and (IPW HR 3.88, 95% CI 1.95 to 7.74). Men exhibited a higher mortality risk at all frailty levels only in unweighted analysis. Country-specific differences were not significant.

**Conclusions:**

A CGA-based FI is an independent predictor of all-cause and cardiovascular mortality, with even mild frailty increasing the risk. Implementing frailty assessments can improve health risk prediction in older adults from Eastern Europe.

**WHAT IS ALREADY KNOWN ON THIS TOPIC**

- Previous studies suggested frailty as a robust predictor of mortality in older populations.
- Differences in baseline characteristics and risk factor distribution between frail and non-frail individuals might affect the validity of the association with mortality.
- No prior studies have explored mortality risk in relation to frailty levels in Eastern Europe, a region with high mortality due to economic and political crises.

**WHAT THIS STUDY ADDS**

- Using two levels of adjustments, including confounder-adjusted models and inverse probability weights, this study found a strong association between all-cause and cardiovascular disease mortality and the degree of frailty status, measured by the Comprehensive Geriatric Assessment-Frailty Index (FI) tool.
- This association was observed even in prefrail individuals and those with mild frailty.
- Adjusted mortality risks by FI were comparable in men and women, while country-specific differences were non-significant.

**HOW THIS STUDY MIGHT AFFECT RESEARCH, PRACTICE OR POLICY**

- This study provides unique information on trend in mortality risk across various levels of frailty in populations where the predictive power of frailty may differ from that reported in the West.
- The relationship was consistent across countries with distinct socioeconomic and mortality risk profile.
- The frailty assessment should be implemented into the risk prediction systems.

## Background

 The world’s population is rapidly ageing, and the burden on healthcare systems is expected to increase, particularly in low-income and middle-income countries including Eastern Europe.^1 2^ Given this trend, identification of potential predictors of mortality in this region is pivotal. Frailty is a common condition among the older people, and its association with increased mortality and hospitalisation risk is well established.^3^,^5^ Decline in physical and cognitive performance and an increased susceptibility to stressors are the hallmarks of frailty.^6 7^ Providing its multidimensional concept, frailty can be measured using various frailty indexes (FI).^5 8 9^

The Rockwood and Mitnitski model, based on the number of deficits across multiple domains, has shown high predictive value for adverse health outcomes and mortality.^10^,^12^ The adapted version based on Comprehensive Geriatric Assessment (CGA-FI) is the most commonly used in clinical practice.^9 10^ Other types of FI include the Physical Frailty Phenotype and the Clinical Frailty Scale perform well in predicting health outcomes but are less generalisable compared with the CGA-FI.^9 10^

Eastern and Western European countries differ significantly in demographic profiles, healthcare systems and economic development. Eastern European populations have higher mortality rates due to socioeconomic disparities and distinct lifestyle habits, such as low physical activity and increased tobacco and alcohol consumption,^13^ but there is less evidence on health and mortality of older persons. Studies based on the Study on Health, Ageing and Retirement in Europe (SHARE) data compared the distribution of FI between European countries revealed higher FI and mortality rates in Eastern Europe.^14 15^ However, these results were derived using different frailty measurement approaches and might not be reliable. Given the rapid population ageing, more studies are needed to explore the predictive value of frailty in terms of mortality in these countries.

The prospective Health, Alcohol and Psychosocial factors in Eastern Europe (HAPIEE) cohort study aims to investigate risk factors for high rates of mortality and cardiovascular diseases (CVDs) in Eastern European countries (eg, Czech Republic, Poland and Lithuania).^16^ To our knowledge, previous studies have not evaluated the risk of death associated with the degree of frailty based on the CGA-FI in these populations. Moreover, differences in baseline characteristics and risk factor distribution between frail and non-frail people might affect the validity of mortality assessment. In this study, we aimed to investigate the trend in frailty in association with all-cause and cardiovascular mortality in the Eastern European populations in terms of level of frailty after balancing potential confounders between the comparison groups. Understanding a dose-response effect of frailty enables targeted interventions to reduce mortality risk in older adults.

## Methods

### Study design and participants

Data from the multinational HAPIEE Project were used in this study.^16^ The study recruited 26 746 randomly selected individuals from population registers in selected urban centres in the Czech Republic, Poland and Lithuania, with a mean age of 59±7.3 years old. Baseline data on factors included in this study such as age, sex, health status, medical examinations, lifestyle, socioeconomic status and psychosocial factors were collected between 2002 and 2005 in the Czech Republic and Poland and between 2005 and 2008 in Lithuania (when it joined the study) using face-to-face computer-assisted personal interviews combined with clinical examinations. The study included individuals with complete follow-up data until the end of 2020 in the Czech Republic, until 31 July 2017 in Poland and until 31 March 2019 in Lithuania.

### Measurement of FI (CGA-FI)

**Figure 1:**
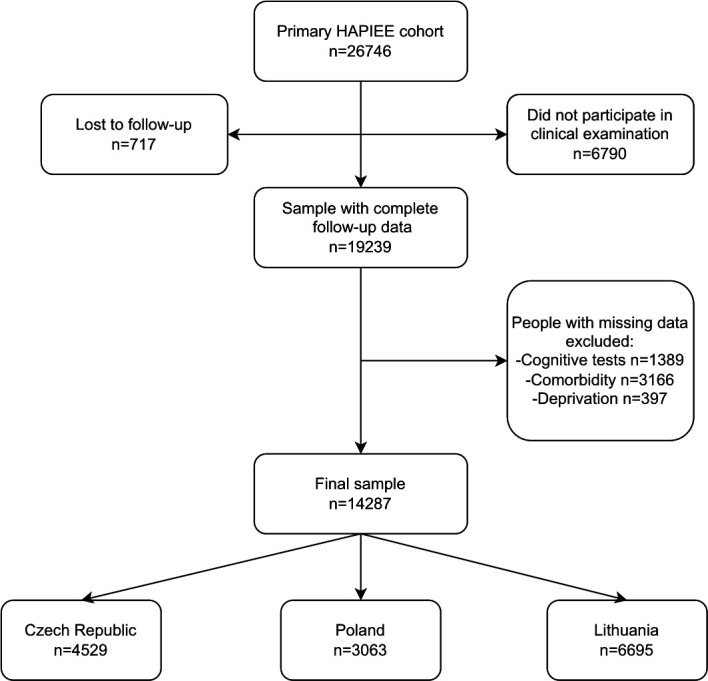
Flow chart of exclusion criteria of the HAPIEE study cohort. HAPIEE, Health, Alcohol and Psychosocial factors in Eastern Europe.

The CGA-FI uses 41 items. In the HAPIEE cohort, complete data were available for 21 items (figure 1). FI with over 10 items is an accepted valid predictor of health outcomes.^10 17^ All binary variables were coded as ‘1’ indicating a presence of deficit and ‘0’ otherwise. The following variables were included in the calculation of CGA-FI:

1. Self-reported comorbidities: stroke, myocardial infarction, ischaemic heart disease, hypertension (defined as measured blood pressure >140/90 mm Hg and/or self-reported treated hypertension), diabetes (treated and/or untreated), asthma and chronic obstructive pulmonary disease (COPD) and depression. Depressive symptoms were measured using Centre for Epidemiologic Studies Depression Scale (CES-D) based on 10 items in Lithuania and 20 items in the Czech Republic and Poland. People with a score equal to or above 16 for CES-D 20 and 4 for CES-D 10 are considered as having a depression.^18 19^
2. Functional activity: loss of independence in activities daily living (ADL) with six items including feeding, dressing, bed mobility, toileting, cooking and shopping and instrumental ADL with four items including telephoning, taking medications, managing finances and picking up small objects.
3. Physical functioning was assessed by the grip strength. Grip strength was measured with the Scandidact handgrip dynamometer twice on the dominant hand for each participant, and the highest value of the two measurements was used in the analysis.^16^ Grip strength was then categorised into three levels of impairment based on sex-specific cut-off points with 0 (no impairment), 0.5 (mild impairment) and 1.0 (major impairment). The following cut-off points were considered: 0—men≥33 kg and women≥21; 0.5—men 33–26 kg and women 21–16 kg; 1 men <26 and women <16 kg.
4. Cognitive functioning was assessed with the use of four standardised tests that were adopted from the Consortium to Establish a Registry for Alzheimer’s Disease including immediate and recall verbal memory using a 10-word learning test, verbal fluency that was measured by asking to name as many animals in 1 min and speed and concentration that was assessed by asking participants to cross out as many letters (‘P’ and ‘W’) in 1 min. All measures were further transformed into standardised z-scores with a mean of 0 and an SD of 1. The average of z-scores from all tests was combined to create the composite score of cognitive function. The composite score was further divided into quartiles to account for the level of cognitive impairment and scored quartiles as 0, 0.3, 0.7 and 1.0 points from highest to lowest, respectively. The distribution of the composite score in terms of age and sex is shown in online supplemental figure 1.
5. Nutritional status was assessed as body mass index <21 kg/m^2^ calculated using the weight and height measured at the medical examination.

FI for each person was calculated as the proportion of number of deficits to the total number of items evaluated.

The calculated FI ranged from 0 to 1, with higher values indicating a greater degree of frailty. The following frailty categories were used: robust (<0.15), prefrailty (0.15 to <0.25), mild frailty (0.25 to <0.35), moderate frailty (0.35 to <0.45), severe frailty (0.45 to <0.55) and advanced frailty (0.55 or higher). Due to the limited number of participants in the advanced category, it was merged with the severe category for further analysis. Sensitivity analysis including all categories is presented in online supplemental table 5.

The selection of items, scoring scale and corresponding cut-off points was based on the official CGA-FI tool, otherwise known as the ‘Senior Health Calculator’^20^ that demonstrates a good predictive performance in mortality assessment (https://www.bidmc.org/research/research-by-department/medicine/gerontology/calculator).^21^ Online supplemental figure 2 displays the distribution of CGA-FI with regard to age and sex.

### Outcome

The main objective was to assess all-cause mortality. Dates of death were obtained from the national death registers in each country. All registers have been shown a complete coverage of deaths.^16^ To examine deaths related to CVD, only deaths restricted to diseases of the circulatory system (International Classification of Diseases Version 10 (ICD-10) codes I*) were considered. Participants were censored on the date of death or the end of the study depending on data availability for each country.

### Covariates

Data on covariates were obtained from questionnaires and medical examinations. The selection of variables was based on their known association with mortality.^22 23^ For the adjustment, we considered age, sex, education (primary, secondary education, college or university degree), occupation (employed, retired or unemployed), deprivation scale (graded from 1 as the least deprived up to 12 as the most deprived), smoking status (never, current or past smoker), alcohol consumption (never, graduated frequency from 1 to 3 drinks monthly or 1–5 drinks weekly), physical activity (as number of hours demanding physical activity per week) and healthy diet score.

### Statistical analyses

All analyses were performed with Stata (V.17; StataCorp). Descriptive statistics are presented as means with SD or frequencies with proportions.

The risk of death in association with frailty status was estimated using Cox proportional hazards regression models. FI was categorised into five levels, with the ‘robust’ category serving as the reference. We used a robust variance estimator to account for possible interactions between groups and multiple comparison. Proportional hazards assumptions were confirmed by exploring the parallelism of estimated survival curves for each covariate (online supplemental figure 4–6). HRs with their corresponding 95% CI were estimated by crude and confounder-adjusted models.

Baseline characteristics and the distribution of risk factors between frail and non-frail individuals could affect the outcomes. To address this, Cox models were weighted using inverse probability weights (IPWs) based on propensity scores. Variables for the IPW were selected based on their potential association with the outcome and exposure. Logistic regression was used to determine the propensity score, representing the likelihood of frailty given the baseline covariates. Standardised differences between frail and non-frail individuals were compared before and after IPW application to assess balance, with differences above 0.10 considered potentially meaningful (tables1^2^, online supplemental table 1–3 and figure 3).^24 25^

**Table 1:** Characteristics of the study sample of people according to frailty status (n=14 287)

Variables	Non-frail(n=13 396)		Frail*(n=891)		Standardised difference (Cohen’s d)
Age (years), mean (SD)	59.3	7.4	62.8	6.5	0.5
Age (years), n (%)					0.5
<50	1965	(14.7)	35	(3.9)	
50–59	4924	(36.8)	240	(26.9)	
60–69	5639	(42.1)	502	(56.3)	
≥70	868	(6.5)	114	(12.8)	
Sex, n (%)					0.1
Males	6228	(46.5)	352	(39.5)	
Females	7168	(53.5)	539	(60.5)	
Country, n (%)					0.3
Czech Republic	4344	(32.4)	185	(20.8)	
Poland	2852	(21.3)	211	(23.7)	
Lithuania	6200	(46.3)	495	(55.6)	
Education, n (%)					0.5
Incomplete	37	(0.3)	11	(1.2)	
Primary	1267	(9.5)	189	(21.3)	
Vocational	2538	(19.0)	189	(21.3)	
Secondary	4336	(32.5)	268	(30.1)	
College	1541	(11.5)	111	(12.5)	
University	3641	(27.3)	121	(13.6)	
Occupational status, n (%)					0.8
Employed	5660	(42.5)	108	(12.2)	
Retired/employed	1709	(12.8)	73	(8.2)	
Retired/unemployed	5363	(40.3)	619	(69.9)	
Unemployed	583	(4.4)	86	(9.7)	
Smoking status, n (%)					0.2
Never	7130	(53.5)	505	(56.9)	
Past smoker	3129	(23.5)	236	(26.6)	
Current smoker	3063	(23.0)	147	(16.6)	
Alcohol consumption†, n (%)					0.5
Never	4096	(31.0)	484	(54.9)	
<1/monthly	3475	(26.3)	169	(19.2)	
1–3/monthly	2807	(21.2)	116	(13.2)	
1–4/weekly	2161	(16.3)	85	(9.6)	
≥5/weekly	692	(5.2)	28	(3.2)	
Deprivation range‡, mean (SD)	1.3	2.2	2.3	2.9	0.4
Physical activity§, mean (SD)					
Moderate	15.1	11.2	12.3	11.8	0.2
Vigorous	4.0	5.3	3.2	5.5	0.2

* Frail status: frail (CGA-FI>0.25).

† Alcohol consumption (never, graduated frequency from 1 to 3 drinks monthly or 1–5 drinks weekly).

‡ Deprivation scale (graded from 1 as athe least deprived up to 12 as athe most deprived).

§ Number of hours of moderate and vigorous physical activity per week.

CGA-FIComprehensive Geriatric Assessment-based Frailty Index

**Table 2:** Characteristics of the study sample of people according to sex (n=14 287)

Variables	Males (n=6580)		Females (n=7707)		Standardised difference (Cohen’s d)
Age (years), mean SD	59.8	7.3	59.3	7.4	0.0
Age (years), n (%)					0.0
<50	862	(13.1)	1 138	(14.8)	
50–59	2370	(36.0)	2 794	(36.3)	
60–69	2880	(43.8)	3 261	(42.3)	
≥70	468	(7.1)	514	(6.7)	
Country, n (%)					0.0
Czech Republic	2038	(31.0)	2 491	(32.3)	
Poland	1501	(22.8)	1 562	(20.3)	
Lithuania	3041	(46.2)	3 654	(47.4)	
Education, n (%)					0.3
Incomplete	20	(0.3)	28	(0.4)	
Primary	548	(8.4)	908	(11.8)	
Vocational	1490	(22.7)	1 237	(16.1)	
Secondary	2007	(30.6)	2 597	(33.8)	
College	598	(9.1)	1 054	(13.7)	
University	1896	(28.9)	1 866	(24.3)	
Occupational status, n (%)					0.2
Employed	2953	(45.2)	2 815	(36.7)	
Retired/employed	935	(14.3)	847	(11.1)	
Retired/unemployed	2361	(36.1)	3 621	(47.3)	
Unemployed	289	(4.4)	380	(5.0)	
Smoking status, n (%)					0.7
Never	2357	(36.0)	5 278	(68.8)	
Past smoker	2250	(34.4)	1 115	(14.5)	
Current smoker	1934	(29.6)	1 276	(16.6)	
Alcohol consumption*, n (%)					0.8
Never	1079	(16.6)	3 501	(46.1)	
<1/monthly	1593	(24.4)	2 051	(27.0)	
1–3/monthly	1714	(26.3)	1 209	(15.9)	
1–4/weekly	1535	(23.5)	711	(9.4)	
≥5/weekly	598	(9.2)	122	(1.6)	
Deprivation range†, mean (SD)	1.1	2.0	1.6	2.4	0.2
Physical activity‡, mean (SD)					
Moderate	13.1	10.8	16.5	11.5	0.3
Vigorous	4.0	5.4	3.9	5.2	0.0
Frailty related variables					
Frailty index, mean (SD)	0.13	0.1	0.12	0.1	0.0
BMI, mean (SD), kg/m^2^	28.4	4.2	29.1	5.4	0.1
Grip strength, mean (SD), kg	46.1	9.2	28.3	6.5	2.3
Global cognitive Z-score§, mean (SD)	−0.2	0.7	0.1	0.7	0.4
Comorbidities, n (%)					
Cardiovascular diseases					
Hypertension	4626	(70.6)	4 495	(58.5)	0.3
Myocardial infarction	614	(9.4)	318	(4.2)	0.2
Ischaemic heart disease	667	(10.3)	870	(11.5)	0.0
Stroke	251	(3.9)	232	(3.1)	0.0
Lung diseases					
COPD	884	(13.6)	1141	(15.1)	0.0
Asthma	247	(3.8)	415	(5.5)	0.0
Other diseases					
Diabetes	661	(10.1)	666	(8.7)	0.0
Depression	4059	(61.7)	5 174	(67.1)	0.1

Frail status: frail (CGA-FI>0.25).

* Alcohol consumption (never, graduated frequency from 1 to 3 drinks monthly or 1–5 drinks weekly).

† Deprivation scale (graded from 1 as athe least deprived up to 12 as athe most deprived).

‡ Number of hours of moderate and vigorous physical activity per week.

§ Age-specific, sex-specific and -country -specific composite Z-score of all cognitive tests including episodic memory, verbal fluency and mental speed and concentration tests.

BMI, body mass index; CGA-FIComprehensive Geriatric Assessment-based Frailty IndexCOPD, chronic obstructive pulmonary disease

The main analysis included the entire sample. Stratified analysis by sex and country was conducted to account for differences in baseline characteristics. The mean follow-up ranged from 10.9 years in Lithuania to 15.7 years in the Czech Republic. To assess whether this variation affects the results, we conducted a sensitivity analysis with a restricted follow-up to 10 years.

## Results

Among 26 746 individuals recruited in the cohort, 14 287 had complete data and satisfied the inclusion criteria, 6580 (46%) men and 7707 (54%) women (figure 1). The relatively large number of persons with missing data was mainly due to the low-response rate in Czech and Polish participants in attending the clinical examination which was conducted on another day than the interview. People with frailty were older with a greater proportion of women, higher rates of retirement and unemployment, as well as lower levels of education attainment and physical activity (table 1). These differences were balanced after applying IPW.

In a comparative analysis of sex-related differences, men had a higher prevalence of smoking, heavy drinking, along with a higher frequency of cardiovascular comorbidities and lower cognitive assessment scores (table 2). Conversely, women had higher rates of unemployment, deprivation and following comorbidities including asthma, diabetes and depressive symptoms (table 2).

When looking at differences between countries, individuals from the Czech Republic had a higher rate of alcohol use (online supplemental table 4). In terms of comorbidities, Poland showed a higher prevalence of ischaemic heart disease, myocardial infarction and diabetes while COPD with respiratory symptoms was more frequent among the Lithuanian population.

During an average follow-up period of 13 years, 2402 deaths occurred (online supplemental tables 1 and 2). Mortality rate increased progressively with the degree of frailty status, ranging from 1.95 (95% CI 1.81 to 2.10) to 4.86 (95% CI 3.73 to 6.33) per 100 person-years for prefrail and severely frail individuals compared with non-frail people (mortality rate 1.00, 95% CI 0.93 to 1.03 per 100 person-years) (table 3). Unadjusted Cox proportional hazards regression analysis showed a more than twofold increased risk of death for mild frailty (HR 2.81, 95% CI 2.42 to 3.27) and a more than fivefold increased risk for severe frailty (HR 5.58, 95% CI 4.22 to 7.39) compared with robust frailty. Adjusting for baseline covariates reduced the HR for mild and severe frailty to 1.84 (95% CI 1.56 to 2.17) and 2.97 (95% CI 2.21 to 3.99), respectively. After applying IPW, mild (IPW HR 2.06, 95% CI 1.60 to 2.66) and severe (IPW HR 2.71, 95% CI 1.45 to 5.07) frailty groups had more than two times elevated risk of death compared with non-frail individuals from the robust frailty category (table 3).

**Table 3:** Association between degree of frailty and all-cause and CVD mortality

Frailty categories*	No. of persons	No. of deaths	Person-years of follow-up	Deaths per 100 person-years (95% CI)	Unadjusted HR (95% CI)	Adjusted HR† (95% CI)	IPW HR‡ (95% CI)
All-cause mortality							
Robust	10 556	1373	140 104	1.00 (0.93 to 1.03)	1.00	1.00	1.00
Prefrail	2840	711	36 520	1.95 (1.81 to 2.10)	2.01 (1.83 to 2.19)	1.46 (1.32 to 1.62)	2.02 (1.84 to 2.22)
Mild	612	189	7216	2.62 (2.27 to 3.02)	2.81 (2.42 to 3.27)	1.84 (1.56 to 2.17)	2.06 (1.60 to 2.66)
Moderate	165	74	1794	4.12 (3.28 to 5.18)	4.55 (3.57 to 5.80)	2.97 (2.31 to 3.82)	2.25 (1.39 to 3.64)
Severe	114	55	1132	4.86 (3.73 to 6.33)	5.58 (4.22 to 7.39)	2.97 (2.21 to 3.99)	2.71 (1.45 to 5.07)
CVD mortality							
Robust	10 556	482	140 104	0.34 (0.31 to 0.38)	1.00	1.00	1.00
Prefrail	2840	310	36 520	0.85 (0.76 to 0.95)	2.50 (2.17 to 2.89)	1.72 (1.47 to 2.02)	2.50 (2.15 to 2.91)
Mild	612	98	7216	1.36 (1.11 to 1.66)	4.14 (3.33 to 5.14)	2.49 (1.94 to 3.19)	3.05 (2.14 to 4.35)
Moderate	165	39	1794	2.17 (1.59 to 2.98)	6.78 (4.88 to 9.42)	3.80 (2.64 to 5.48)	4.15 (2.35 to 7.33)
Severe	114	29	1132	2.56 (1.78 to 3.68)	8.29 (5.71 to 12.0)	3.96 (2.62 to 5.98)	3.88 (1.95 to 7.74)

* Frailty categories based on a Comprehensive Geriatric Assessment Frailty Index (CGA-FI).

† Adjusted for age, sex, country, occupation, education and deprivation level; alcohol consumption, smoking status and level of physical activity.

‡ Inverse probability weighting based on propensity score derived from characteristics listed in Supplementary Table 1online supplemental table 1.

CVDcardiovascular diseaseIPW HRinverse probability weighting HR

When the analysis was restricted to CVD mortality, the risk of death was more than threefold increased for mild (IPW HR 3.05, 95% CI 2.14 to 4.35) and severe (IPW HR 3.88, 95% CI 1.95 to 7.74) frailty, and the greatest increased risk of death was observed in individuals with moderate frailty (HR 4.15, 95% CI 2.35 to 7.33) (table 3).

Analysis stratified by sex showed a similar trend in both men and women, with a stronger association between frailty and mortality in men in unadjusted models (HR 6.15, 95% CI 4.33 to 8.73) compared with women (HR 5.51, 95% CI 3.50 to 8.67) in the severe frailty group (table 4). However, sex differences were almost non-existent in weighted regression models.

**Table 4:** Association between degree of frailty and all-cause mortality by sex

Frailty categories*	No. of persons	No. of deaths	Person-years of follow-up	Deaths per 100 person-years (95% CI)	Unadjusted HR (95% CI)	Adjusted HR† (95% CI)	IPW HR‡ (95% CI)
Males							
Robust	4879	859	62 940	1.36 (1.28 to 1.46)	1.00	1.00	1.00
Prefrail	1349	456	16 816	2.71 (2.47 to 2.97)	2.00 (1.79 to 2.24)	1.58 (1.39 to 1.79)	1.98 (1.76 to 2.23)
Mild	257	109	2940	3.71 (3.07 to 4.47)	2.83 (2.32 to 3.44)	2.03 (1.62 to 2.54)	2.06 (1.49 to 2.86)
Moderate	47	32	445	7.19 (5.08 to 10.2)	5.75 (3.87 to 8.55)	3.66 (2.40 to 5.57)	3.10 (1.51 to 6.37)
Severe	48	33	446	7.41 (5.27 to 10.4)	6.15 (4.33 to 8.73)	3.25 (2.20 to 4.81)	2.51 (1.00 to 6.55)
Females							
Robust	5677	514	77 164	0.67 (0.61 to 0.73)	1.00	1.00	1.00
Prefrail	1491	255	19 704	1.29 (1.14 to 1.46)	1.97 (1.70 to 2.29)	1.32 (1.12 to 1.55)	2.01 (1.72 to 2.36)
Mild	355	80	4276	1.87 (1.50 to 2.33)	3.01 (2.38 to 3.81)	1.70 (1.31 to 2.20)	1.90 (1.24 to 2.92)
Moderate	118	42	1349	3.11 (2.30 to 4.21)	5.14 (3.74 to 7.08)	2.62 (1.88 to 3.64)	2.49 (1.35 to 4.59)
Severe	66	22	687	3.20 (2.11 to 4.86)	5.51 (3.50 to 8.67)	2.83 (1.75 to 4.58)	2.54 (1.29 to 5.00)

* Frailty categories based on a Comprehensive Geriatric Assessment Frailty Index (CGA-FI).

† Adjusted for age, country, occupation, education and deprivation level; alcohol consumption, smoking status and level of physical activity.

‡ Inverse probability weighting based on propensity score derived from characteristics listed in Supplementary Table 1online supplemental table 1.

IPW HRinverse probability weighting HR

There were no significant differences in the risk of death associated with frailty between countries (data are not shown). However, the CIs were wider and the differences in HRs between countries were not statistically significant (p=0.08). Similar results were obtained with a balanced follow-up time (restricted to 10 years) (online supplemental table 6).

## Discussion

This study examined the relationship between frailty, measured by the CGA-FI tool, and all-cause and CVD mortality in older adults in three Eastern European countries. Our results demonstrated a strong association of all-cause and CVD mortality with the degree of frailty status, even after balancing comparison groups. Mortality risk in men was higher across all levels of frailty but only in unweighted analysis. Country-specific variations were non-significant.

Frailty is more prevalent among older individuals in Eastern Europe than in Western countries, with a pooled prevalence of around 10%, which may be attributed to poorer socioeconomic conditions and higher rates of chronic diseases.^26 27^ The proportion of frail people in our study was about 6%, likely due to different measurements used to assess frailty. The findings from the SHARE study identified the Czech Republic and Poland as the countries with the highest CGA-FI mean score, ranging from 0.17 to 0.24.^14^ These figures were higher than those observed in our cohorts (the means ranged from 0.11 to 0.14 across three countries with the highest frailty seen in Poland). The lower prevalence of CGA-FI in our study might be explained by different set of parameters included in CGA-FI calculations.

Previous research has shown that CGA-FI is linked to an increased risk of mortality in older adults with HRs ranging from 1.8 to 2.3 across countries.^28^,^30^ The study on community-dwelling people aged 60 and over found an increased all-cause mortality risk in prefrail (HR 1.65, 95% CI 1.45 to 1.85) compared with frail individuals (HR 2.79, 95% CI 2.35 to 3.30).^31^ Our findings are consistent with these results, with mild and severe frailty associated with HRs of 1.84 (95% CI 1.56 to 2.17) and 2.97 (95% CI 2.21 to 3.99), respectively, in the adjusted model. The slightly stronger associations in our study may be due to the use of CGA-FI scores rather than an adapted version of Fried’s frailty criteria used in other studies. Our study also confirmed a stronger association between frailty and CVD mortality, with HRs of 3.05 (95% CI 2.14 to 4.35) and 3.88 (95% CI 1.95 to 7.74) for mild and severe frailty, respectively.^31^ Similar findings were reported by other studies using the continuous scale of CGA-FI.^4 12^ ^32^ The stronger association between frailty and CVD mortality may be explained by factors such as chronic inflammation, immune dysregulation, sarcopenia and cardiovascular risk factors.^33 34^

Sex differences in frailty and mortality risk were consistent with previous studies, showing a higher prevalence of frailty in women but higher mortality risk in men associated with frailty.^35^,^37^ Similarly to our findings, these results were observed only in unadjusted models.^4 32 36 37^ One possible explanation is that women may have higher baseline functional reserve, which delays adverse outcomes while men may develop frailty due to CVD, which is associated with higher mortality risk.^38^

### Strengths and limitations

This prospective cohort study provides unique information on populations from Eastern Europe. The large number of investigating covariates to be adjusted in the analyses of frailty and mortality is the particular strength of this study. The response rate was comparable and follow-up time was balanced between countries.

It is important to consider the limitations of our study. First, the study sample was restricted to specific urban populations, so it does not include rural areas, which may limit the generalisability of our findings to other populations. Second, the study relied, to some extent, on self-reported data, which may introduce recall bias. Third, a relatively large number of Czech and Polish participants with missing data were less healthy and did not undergo the baseline clinical examination, which could have led to an underestimation of the prevalence of frailty and may have affected the estimates (online supplemental table 7). Thus, the assumption of missing at random was violated, we opted against using multiple imputation for handling missing data.

Fourth, the study did not have information on all parameters ideally required for calculating the frailty score, which may affect the precision and stability of the results. However, it has been assumed that this should not impact the accuracy of predicted outcomes as long as the number of parameters exceeds a certain low limit (min 10).^10^ It has also been shown that the reduced number of deficits may simplify the FI calculation without reducing its validity.^39^

Fifth, a number of people in the severe frailty groups were too small for investigations with sufficient statistical power, particularly in sex-specific analysis. Therefore, the comparison results in these groups should be interpreted with caution.

We estimated frailty using the CGA-FI, a widely accepted and validated method for assessing frailty in older people.^9^ This index has been shown to have comparable performance with other prognostic models in predicting mortality and other adverse outcomes.^20^ It has been used in various studies, including the SHARE study^14^ which ensures the comparability of the results. Moreover, this tool has been found to be superior to other methods that may not capture the full spectrum of factors contributing to frailty.^9^

The use of IPW as an additional adjustment in this study helped balance baseline differences between frail and non-frail individuals, which could have otherwise led to biased estimates.^24^ This method has been used in a range of other studies including the research on the association between frailty and mortality.^40^

Finally, the nature of our design cannot entirely prove causality, although the longitudinal design, extensive covariate adjustment of models including IPW and long follow-up are likely to minimise these limitations.

## Conclusions

This study confirms a strong association between frailty and higher mortality risk in older adults in Eastern Europe. The results were consistent across countries with different socioeconomic contexts and mortality risk profile and in line with previous research from western countries. Frailty consistently predicted both all-cause and cardiovascular mortality in a dose-response manner, even in individuals with mild frailty. This study supports the inclusion of frailty assessment into risk prediction models. Given the relative paucity of research on ageing in the region, these findings may encourage further investigations of key ageing phenotypes, including frailty.

## supplementary material

10.1136/jech-2023-221761 online supplemental file 1

## Data Availability

Data are available on reasonable request. Data may be obtained from a third party and are not publicly available.
