# Supplementary material for: Frailty index is an independent predictor of all-cause and cardiovascular mortality in Eastern Europe: a multicentre cohort study
Source: J Epidemiol Community Health. 2024 Aug 24;79(1):e221761. doi: 10.1136/jech-2023-221761 (PMC11671974; doi:10.1136/jech-2023-221761)
Supplement: online supplemental file 1 [file jech-79-1-s001.pdf]

Supplementary data

Supplementary Table 1. Characteristics of the study sample of people according to Death Status (n=14 287)

Variables	Dead (n=2 402)		Alive (n=11 885)		Standardized Difference (Cohen's d)
Age (years), mean SD	63.6	6.5	58.7	7.3	0.7
**Age (years), n (%)**					0.7
< 50	93	(3.9)	1 907	(16.0)	
50-59	538	(22.4)	4 626	(38.9)	
60-69	1 404	(58.5)	4 737	(40.0)	
≥ 70	367	(15.3)	615	(5.2)	
**Sex, n (%)**					0.4
Men	1 489	(62.0)	5 091	(42.8)	
Women	913	(38.0)	6 794	(57.2)	
**Country, n (%)**					0.1
Czech Republic	834	(34.7)	3 695	(31.1)	
Poland	412	(17.2)	2 651	(22.3)	
Lithuania	1 156	(48.1)	5 539	(46.6)	
**Education, n (%)**					0.3
Incomplete	23	(1.0)	25	(0.2)	
Primary	399	(16.7)	1 057	(8.9)	
Vocational	513	(21.5)	2 214	(18.7)	
Secondary	765	(32.0)	3 839	(32.4)	
College	230	(9.6)	1 422	(12.0)	
University	460	(19.2)	3 302	(27.8)	
**Occupational status, n (%)**					0.6
Employed	457	(19.2)	5 311	(44.9)	
Retired/employed	303	(12.7)	1 479	(12.5)	
Retired/unemployed	1514	(63.6)	4 468	(37.8)	
Unemployed	106	(4.5)	563	(4.8)	
**Smoking status, n (%)**					0.3
Never	1006	(42.2)	6 629	(56.0)	
Past smoker	692	(29.0)	2 673	(22.6)	
Current smoker	685	(28.7)	2 525	(21.3)	
**Alcohol consumption[b], n (%)**					0.1
Never	800	(33.9)	3 780	(32.2)	
<1/monthly	554	(23.5)	3 090	(26.3)	
1-3/monthly	472	(20.0)	2 451	(20.9)	
1-4/weekly	368	(15.6)	1 878	(16.0)	
≥5/weekly	166	(7.0)	554	(4.7)	
Deprivation range[c], mean (SD)	1.5	2.4	1.3	2.2	0.0
**Physical activity[d], mean (SD)**					
Moderate	13.4	11.5	15.1	11.2	0.2
Vigorous	3.8	5.5	4.0	5.3	0.0
**Frailty related variables**					
Frailty index, mean (SD)	0.17	0.1	0.12	0.1	0.2
BMI, mean (SD), kg/m^2	29.5	5.4	28.6	4.8	0.2
Grip strength, mean (SD), kg	36.3	11.7	36.6	11.9	0.0
Global cognitive Z-score[e], mean (SD)	-0.4	0.8	0.1	0.7	0.2
**Comorbidities, n (%)**					
Cardiovascular diseases					
Hypertension	1 839	(77.2)	7 282	(61.4)	0.4
Myocardial infarction	323	(13.8)	609	(5.2)	0.3
Ischemic heart disease	388	(16.5)	1 149	(9.8)	0.2
Stroke	159	(6.8)	324	(2.8)	0.2

Lung diseases					
COPD	431	(18.4)	1 594	(13.6)	0.2
Asthma	156	(6.7)	506	(4.3)	0.1
Other diseases					
Diabetes	413	(17.3)	914	(7.7)	0.3
Depression	1 562	(65.0)	7 671	(64.5)	0.0

BMI, body mass index; COPD, chronic obstructive pulmonary disease.

Frail status: frail (CGA-FI > 0.25)

[a] Smoking category ((current or past heavy smoker (>30 cigarettes per day), moderate smoker (11 - 29 cigarettes per day), or light smoker (<10 cigarettes per day)).

[b] Alcohol consumption (never, graduated frequency from 1-3 drinks monthly or 1-5 drinks weekly).

[c]Deprivation scale (graded from 1 as a least deprived up to 12 as a most deprived).

[d]Number of hours of moderate and vigorous physical activity per week.

[e]Age-, sex- and -country specific composite Z-score of all cognitive tests including episodic memory, verbal fluency and mental speed and concentration tests.

Supplementary Table 2. Characteristics of the study sample of people according to type of death (n=2 402)

Variables	CVD deaths (n=958)		Other deaths (n=1 444)		Standardized Difference (Cohen's d)
Age (years), mean SD	64.8	5.9	62.9	6.7	0.3
**Age (years), n (%)**					0.3
< 50	20	(2.1)	73	(5.1)	
50-59	171	(17.8)	367	(25.4)	
60-69	580	(60.5)	824	(57.1)	
≥ 70	187	(19.5)	180	(12.5)	
**Sex, n (%)**					0.1
Men	620	(64.7)	869	(60.2)	
Women	338	(35.3)	575	(39.8)	
**Country, n (%)**					0.3
Czech Republic	317	(33.1)	517	(35.8)	
Poland	120	(12.5)	292	(20.2)	
Lithuania	521	(54.4)	635	(44.0)	
**Education, n (%)**					0.2
Incomplete	10	(1.1)	13	(0.9)	
Primary	179	(18.8)	220	(15.3)	
Vocational	213	(22.4)	300	(20.8)	
Secondary	281	(29.5)	484	(33.6)	
College	102	(10.7)	128	(8.9)	
University	166	(17.5)	294	(20.4)	
**Occupational status, n (%)**					0.3
Employed	129	(13.6)	328	(22.9)	
Retired/employed	115	(12.1)	188	(13.1)	
Retired/unemployed	657	(69.4)	857	(59.8)	
Unemployed	46	(4.9)	60	(4.2)	
**Smoking status, n (%)**					0.1
Never	403	(42.6)	603	(42.0)	
Past smoker	292	(30.9)	400	(27.8)	
Current smoker	251	(26.5)	434	(30.2)	
**Alcohol consumption[b], n (%)**					0.1
Never	347	(36.8)	453	(32.0)	
<1/monthly	214	(22.7)	340	(24.0)	
1-3/monthly	187	(19.8)	285	(20.1)	
1-4/weekly	135	(14.3)	233	(16.5)	
≥5/weekly	61	(6.5)	105	(7.4)	
Deprivation range[c], mean (SD)	1.4	2.3	1.5	2.4	0.0
**Physical activity[d], mean (SD)**					
Moderate	13.1	11.8	13.7	11.3	0.0
Vigorous	3.7	5.6	3.8	5.5	0.0
**Frailty related variables**					
Frailty index, mean (SD)	0.18	0.1	0.15	0.1	0.2
BMI, mean (SD), kg/m^2	30.3	5.5	29.0	5.3	0.2
Grip strength, mean (SD), kg	36.4	12.0	36.2	11.6	0.0
Global cognitive Z-score[e], mean (SD)	-0.5	0.8	-0.3	0.8	0.3
**Comorbidities, n (%)**					
Cardiovascular diseases					
Hypertension	783	(82.5)	1 056	(73.6)	0.2
Myocardial infarction	177	(18.9)	146	(10.3)	0.2
Ischemic heart disease	190	(20.3)	198	(14.0)	0.2
Stroke	92	(9.8)	67	(4.8)	0.2
Lung diseases					
COPD	171	(18.3)	260	(18.5)	0.0

Asthma	61	(6.5)	95	(6.7)	0.0
Other diseases					
Diabetes	206	(21.6)	207	(14.4)	0.2
Depression	591	(61.7)	971	(67.2)	0.1

BMI, body mass index; COPD, chronic obstructive pulmonary disease.

Frail status: frail (CGA-FI > 0.25)

[a] Smoking category ((current or past heavy smoker (>30 cigarettes per day), moderate smoker (11 - 29 cigarettes per day), or light smoker (<10 cigarettes per day)).

[b] Alcohol consumption (never, graduated frequency from 1-3 drinks monthly or 1-5 drinks weekly).

[c]Deprivation scale (graded from 1 as a least deprived up to 12 as a most deprived).

[d]Number of hours of moderate and vigorous physical activity per week.

[e]Age-, sex- and -country specific composite Z-score of all cognitive tests including episodic memory, verbal fluency and mental speed and concentration tests.

Supplementary Table 3. **Test on balance of the study sample after applying inverse probability weights***

Variables	Standardized mean		Standardize Variance	
	**raw**	**weighted**	**raw**	**weighted**
Age (years), , mean (SD)	0.50	0.08	0.78	0.85
**Sex, n (%)**				
Men	reference			
Women	0.15	0.01	0.96	1.00
**Country, n (%)**				
Czech Republic	reference			
Poland	0.06	0.03	1.09	0.96
Lithuania	0.18	0.01	0.98	1.00
**Education, n (%)**				
Incomplete	reference			
Primary	0.33	0.02	1.95	1.05
Vocational	0.06	0.05	1.10	1.08
Secondary	0.05	0.03	0.96	0.98
College	0.03	0.04	1.08	0.90
University	0.34	0.00	0.58	1.00
**Occupational status, n (%)**				
Employed	reference			
Retired/employed	0.16	0.05	0.66	1.11
Retired/unemployed	0.63	0.05	0.88	1.02
Unemployed	0.22	0.01	2.17	0.94
**Smoking status, n (%)**				
Never	reference			
Past smoker	0.08	0.11	1.09	1.13
Current smoker	0.17	0.04	0.77	0.95
**Alcohol consumption[a], n (%)**				
Never	reference			
<1/monthly	0.17	0.08	0.80	0.91
1-3/monthly	0.21	0.02	0.70	0.98
1-4/weekly	0.21	0.06	0.62	1.12
≥5/weekly	0.09	0.03	0.64	1.13
Deprivation range[b], , mean (SD)	0.43	0.08	1.79	1.21
**Physical activity[c], , mean (SD)**				
Moderate	0.30	0.24	2.73	1.13
Vigorous	0.15	0.12	1.76	1.09

*Standardized difference balance scale: for means ~ 0, for variance ~ 1

[a]Alcohol consumption (never, graduated frequency from 1-3 drinks monthly or 1-5 drinks weekly).

[b]Deprivation scale (graded from 1 as a least deprived up to 12 as a most deprived).

[c]Number of hours of moderate and vigorous physical activity per week.

Supplementary Table 4. Characteristics of the study sample by country (n=14 287)

	**Czech Republic (n=4 529)**	**Poland (n=3 063)**	**Lithuania (n=6 695)**
Age (years), mean (SD)	58.3 (7.1)	58.1 (6.9)	60.9 (7.6)
Age (years), %			
< 50	16.6	16.2	11.9
50-59	37.7	42.5	31.8
60-69	44.0	39.4	43.3
≥ 70	1.7	1.9	13.0
Women, %	54.2	50.6	54.5
Occupational status, %			
Employed	43.4	35.8	39.7
Retired/employed	8.0	5.9	17.5
Retired/unemployed	45.2	51.9	37.2
Unemployed	3.4	6.4	5.6
Smoking status, %			
Current, ≥ 1 cigarette	23.0	28.3	17.4
Current, < 1 cigarette	2.7	2.0	2.1
Past smoker	29.7	28.2	17.9
Never	44.6	41.5	62.6
Smoking category[a], %			
Light	55.4	43.7	45.6
Moderate	38.2	44.1	45.2
Heavy	6.4	12.2	9.2
Alcohol consumption[b], %			
Never	11.7	35.1	47.4
<1/monthly	25.9	21.8	26.7
1-3/monthly	21.1	0.2	19.9
1-4/weekly	28.5	19.1	5.5
≥5/weekly	12.9	3.8	0.5
Deprivation range[c], mean (SD)	1.6 (2.3)	2.1 (2.9)	1.0 (1.9)
Physical activity moderate[d], mean (SD)	13.7 (12.2)	13.9 (10.6)	15.8 (10.8)
Physical activity vigorous[e], mean (SD)	4.4 (5.4)	5.6 (6.1)	2.9 (4.8)
BMI, mean (SD), kg/m^2	28.2 (4.6)	28.3 (4.6)	29.4 (5.3)
**Comorbidities, %**			
Cardiovascular diseases			
Hypertension	65.2	60.5	66.5
Myocardial infarction	5.0	8.3	7.8
Ischemic heart disease	8.2	19.0	9.8
Stroke	3.3	2.5	4.2
Lung diseases			
COPD	14.4	11.3	16.3
Asthma	4.6	6.8	3.9
Cough (>3 months)	14.0	16.6	14.8
Chest pain (>3 months)	12.5	15.3	15.5
Any type of cancer	6.3	5.1	7.2
Other diseases			
Diabetes	11.4	12.1	7.6
Any type of surgery	2.0	1.9	1.0

BMI, body mass index; COPD, chronic obstructive pulmonary disease.
[a]Smoking category ((current or past heavy (>30 cigarettes per day), moderate (11 - 29 cigarettes per day), or light (<10 cigarettes per day)).

[b]Alcohol consumption (never, graduated frequency from 1-3 drinks monthly or 1-5 drinks weekly).
[c]Deprivation scale (graded from 1 as a least deprived up to 12 as a most deprived).
[d]Number of hours per week undertaken by household domain physical activity (e.g., housework, gardening, maintenance of the house etc).
[e]Number of hours of vigorous physical activity per week (e.g., sports, play games and hiking)

Supplementary figure 1

a. Global cognitive function distribution by sex.

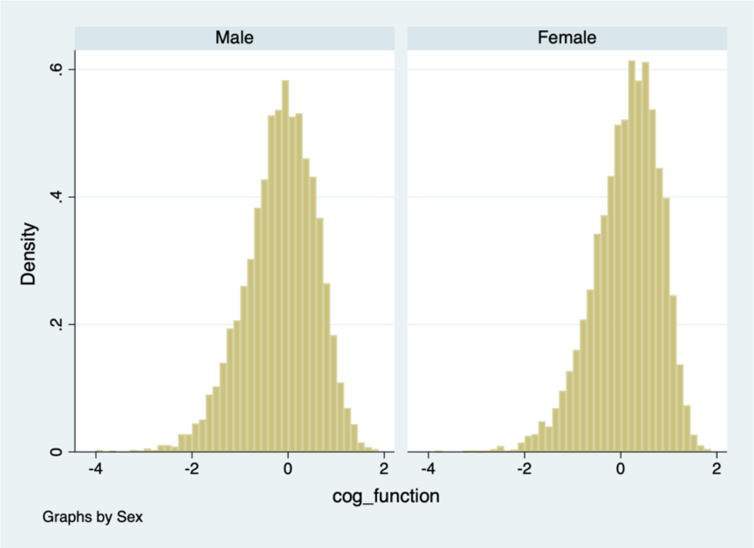

b. Cognitive function quartiles by age and sex.

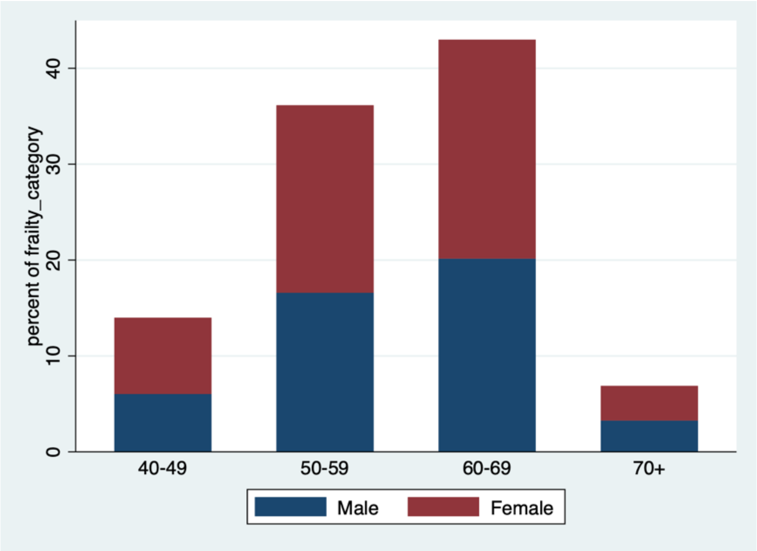

Supplementary figure 2

a. CGA-Fi categories by sex.

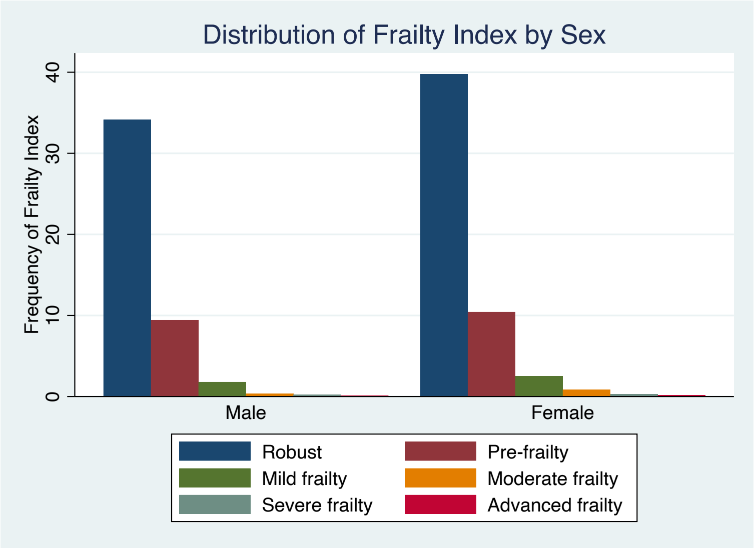

b. CGA-FI categories by age and sex.

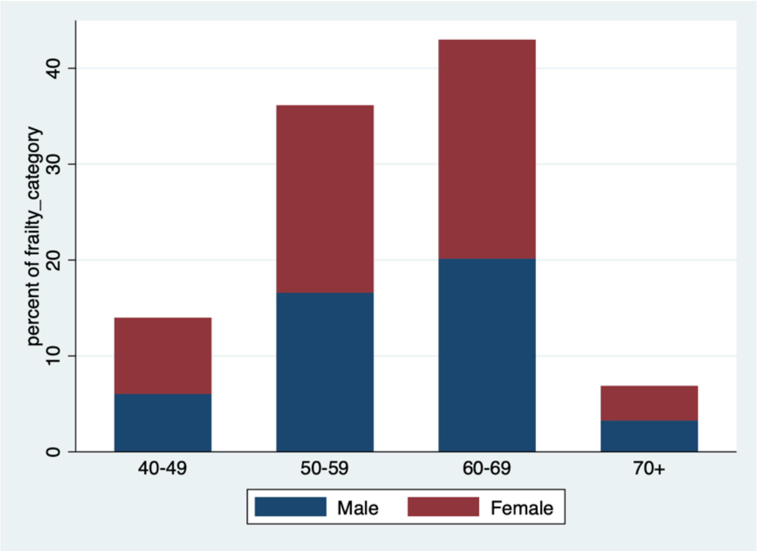

Supplementary figure 3
Check for balance between comparison groups.

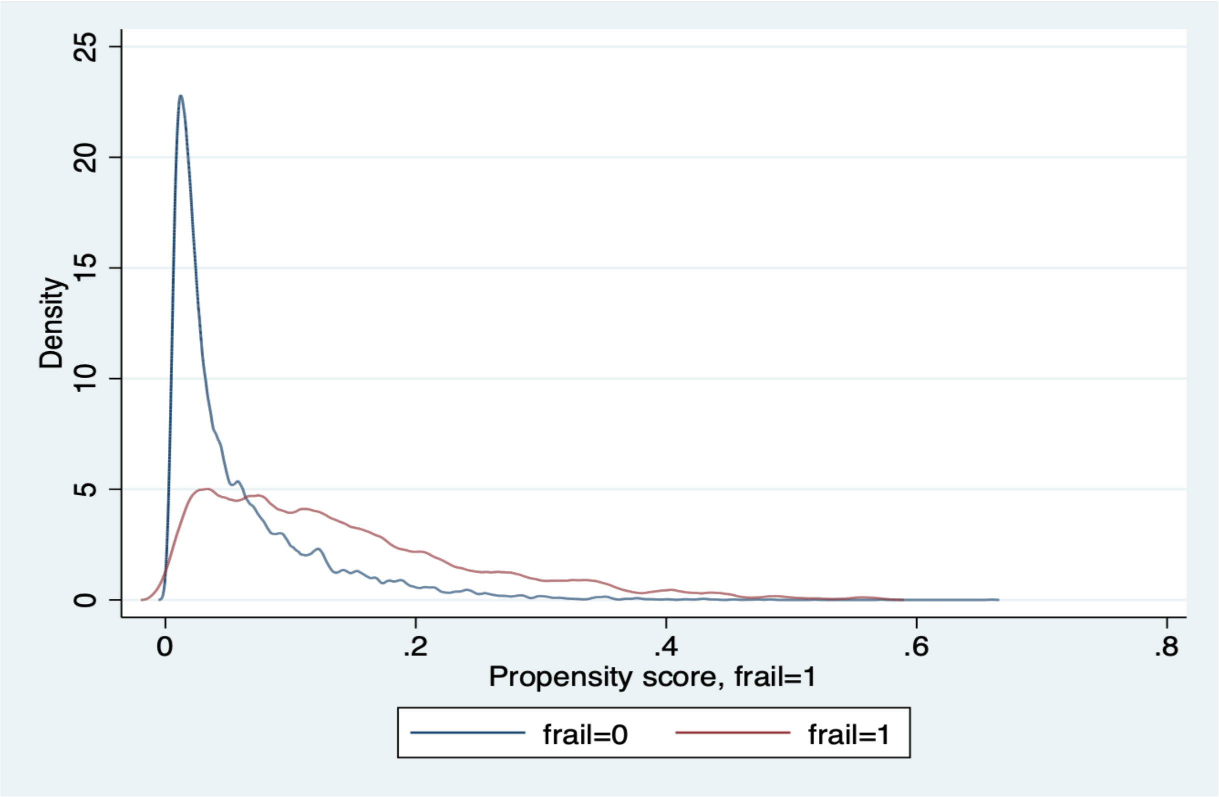

Supplementary Figure 4. KM curves of frailty categories by type of death

a. All-cause mortality

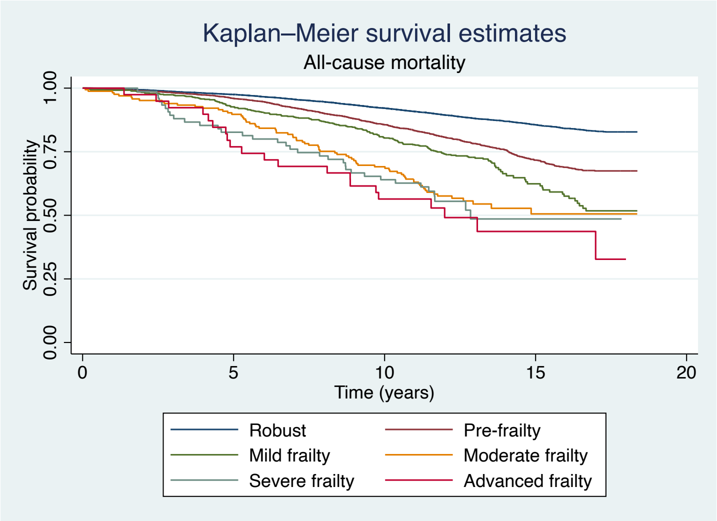

b. CVD mortality

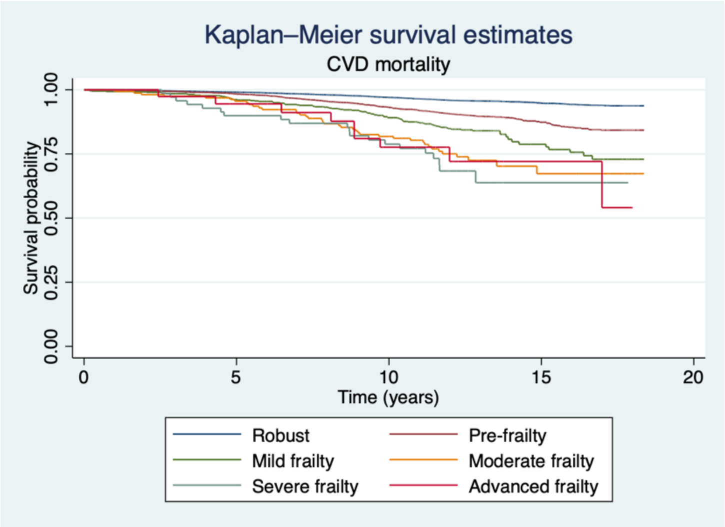

Supplementary Figure 5. KM failure curves of frailty categories by type of death

a. All-cause mortality

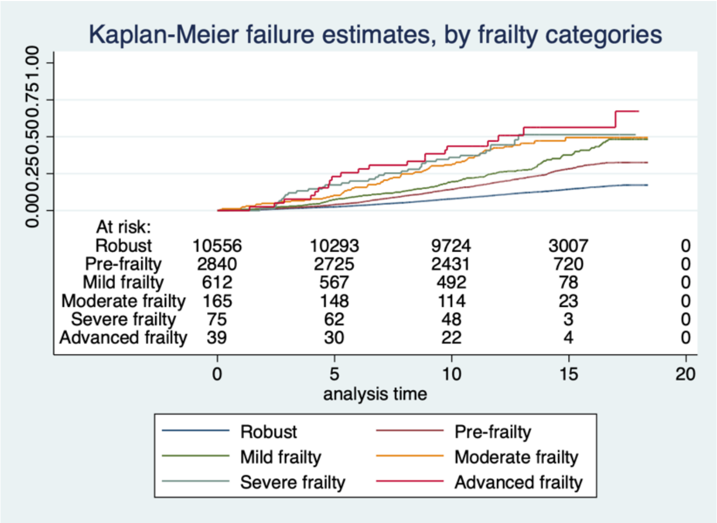

b. CVD mortality

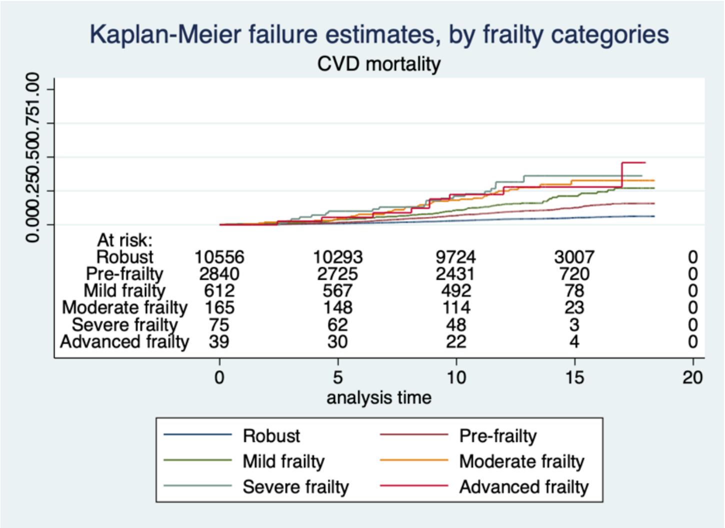

Supplementary Figure 6. KM curves of frailty categories by type of death and sex

a. All-cause mortality

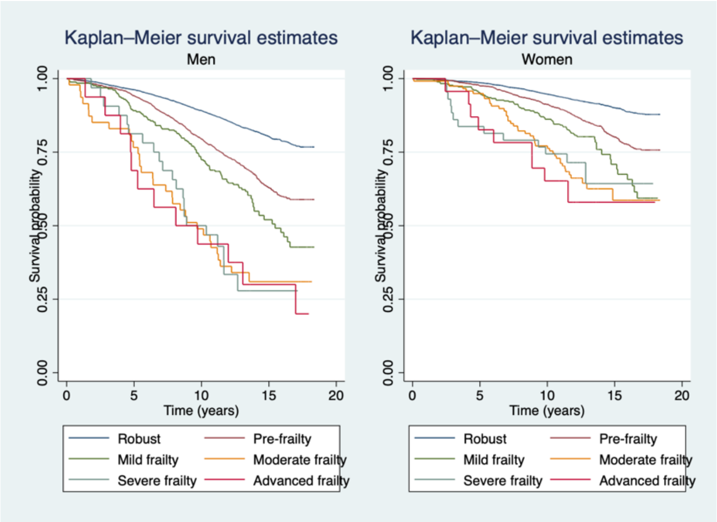

b. CVD mortality

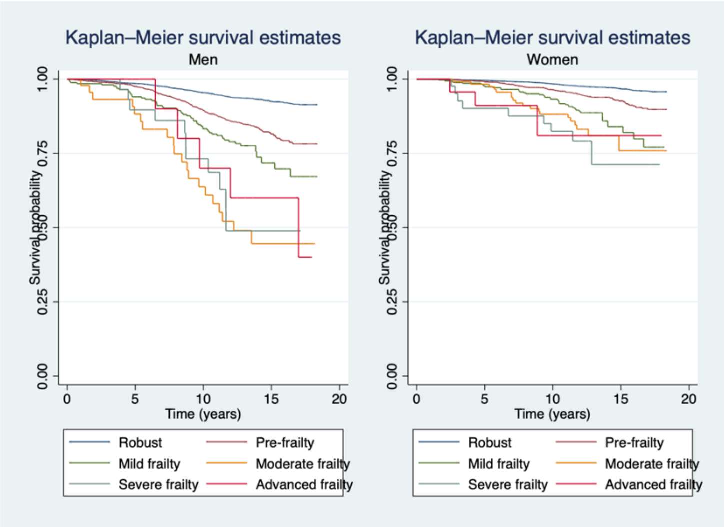

Supplementary Figure 7. Adjusted Cox proportional hazard regression models by frailty categories and type of death

a. All-cause mortality

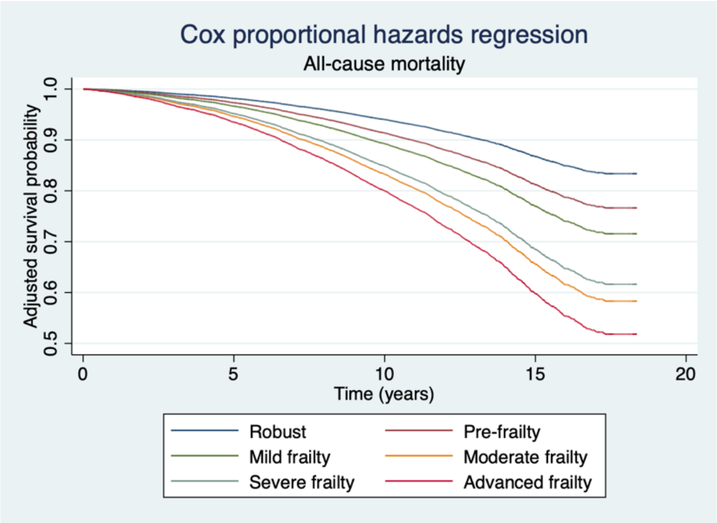

b. CVD mortality

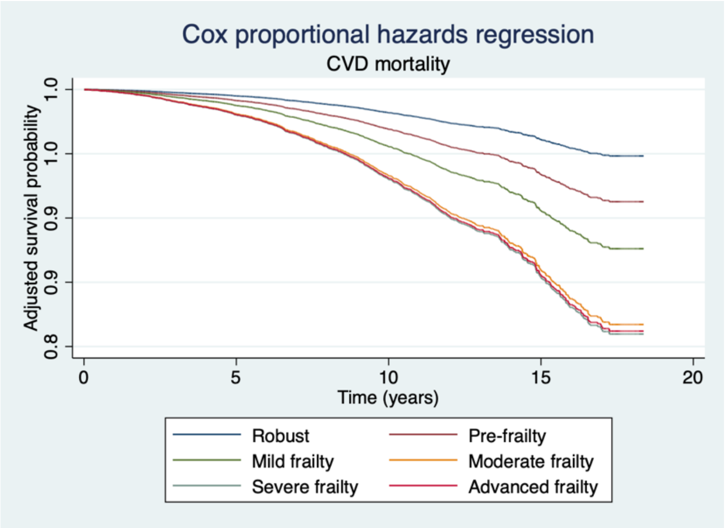

Supplementary Table 5. Association between degree of frailty (with all categories) and all-cause and CVD mortality.

Frailty categories*	No. of persons	No. of deaths	Person-years of follow-up	Deaths per 100 person-years (95% CI)	Unadjusted HR (95% CI)	Adjusted HR† (95% CI)	IPT weighted HR‡ (95% CI)
All-cause mortality							
Robust	10 556	1 373	140 104	1.00 (0.93-1.03)	1.00	1.00	1.00
Pre-frail	2 840	711	36 520	1.95 (1.81-2.10)	2.01 (1.83-2.19)	1.46 (1.32-1.62)	2.02 (1.84-2.22)
Mild	612	189	7 216	2.62 (2.27-3.02)	2.81 (2.42-3.27)	1.84 (1.56-2.17)	2.06 (1.60-2.66)
Moderate	165	74	1 794	4.12 (3.28-5.18)	4.55 (3.57-5.80)	2.97 (2.31-3.81)	2.25 (1.39-3.64)
Severe	75	34	745	4.56 (3.26-6.38)	5.25 (3.69-7.48)	2.66 (1.81-3.92)	3.87 (2.31-6.50)
Advanced	39	21	387	5.42 (3.54-8.32)	6.22 (3.97-9.76)	3.61 (2.37-5.52)	1.66 (0.56-4.94)
CVD mortality							
Robust	10 556	482	140 104	0.34 (0.31-0.38)	1.00	1.00	1.00
Pre-frail	2 840	310	36 520	0.85 (0.76-0.95)	2.50 (2.17-2.89)	1.72 (1.47-2.02)	2.50 (2.15-2.91)
Mild	612	98	7 216	1.36(1.11-1.66)	4.14 (3.33-5.14)	2.49 (1.94-3.19)	3.05 (2.14-4.35)
Moderate	165	39	1 794	2.17 (1.59-2.98)	6.78 (4.88-9.42)	3.80 (2.64-5.48)	4.14 (2.34-7.34)
Severe	75	20	745	2.68 (1.73-4.16)	8.67 (5.55-13.5)	3.97 (2.39-6.60)	5.83 (3.12-10.9)
Advanced	39	9	387	2.32 (1.21-4.47)	7.55 (3.96-14.4)	3.92 (2.05-7.49)	2.16 (0.63-7.40)

CI, confidence interval; HR, hazard ratio.

*Frailty categories based on a Comprehensive Geriatric Assessment Frailty Index (CGA-FI).

†Adjusted for age, sex, country, occupation, education and deprivation level; alcohol consumption, smoking status and level of physical activity.

‡Inverse probability weighting based on propensity score derived from characteristics listed in Supplementary Table 1.

Supplementary Table 6. Association between degree of frailty and all-cause and CVD mortality (follow-up restricted to 10 years).

Frailty categories*	No. of persons	No. of deaths	Person-years of follow-up	Deaths per 100 person-years (95% CI)	Unadjusted HR (95% CI)	Adjusted HR† (95% CI)	IPT weighted HR‡ (95% CI)
All-cause mortality							
Robust	10 556	829	102 283	0.81 (0.76-0.90)	1.00	1.00	1.00
Pre-frail	2 840	409	26 907	1.52 (1.38-1.67)	1.89 (1.68-2.13)	1.47 (1.30-1.67)	1.92 (1.70-2.17)
Mild	612	120	5 653	2.12 (1.77-2.54)	2.66 (2.20-3.22)	1.85 (1.51-2.27)	1.72 (1.29-2.30)
Moderate	165	51	1 429	3.57 (2.71-4.70)	4.58 (3.44-6.10)	2.88 (2.12-3.91)	2.50 (1.58-3.97)
Severe	114	44	944	4.66 (3.47-6.26)	6.08 (4.46-8.28)	3.16 (2.30-4.36)	2.95 (1.76-4.94)
CVD mortality							
Robust	10 556	305	102 283	0.30 (0.27-0.33)	1.00	1.00	1.00
Pre-frail	2 840	186	26 907	0.69 (0.60-0.80)	2.34 (1.95-2.81)	1.71 (1.41-2.09)	2.38 (1.97-2.87)
Mild	612	63	5 653	1.11 (0.87-1.43)	3.81 (2.91-5.00)	2.38 (1.78-3.20)	2.46 (1.71-3.54)
Moderate	165	27	1 429	1.89 (1.30-2.75)	6.64 (4.48-9.85)	3.52 (2.28-5.42)	4.31 (2.40-7.75)
Severe	114	21	944	2.22 (1.45-3.41)	7.96 (5.13-12.4)	3.52 (2.21-5.62)	3.93 (2.10-7.35)

CI, confidence interval; HR, hazard ratio.

*Frailty categories based on a Comprehensive Geriatric Assessment Frailty Index (CGA-FI).

†Adjusted for age, sex, country, occupation, education and deprivation level; alcohol consumption, smoking status and level of physical activity.

‡Inverse probability weighting based on propensity score derived from characteristics listed in Supplementary Table 1

Supplementary table 7. **Characteristics between individuals in the full and analytical sample**

Variables	Full sample (n=26 746)		Analytical sample (n=14 287)	
Age (years), mean SD	59.2	7.4	59.5	7.4
**Age (years), n (%)**				
< 50	4 297	(16.1)	2 000	(14.0)
50-59	10 131	(37.9)	5 164	(36.1)
60-69	11 080	(41.4)	6 141	(43.0)
≥ 70	1 238	(4.6)	982	(6.9)
**Sex, n (%)**				
Men	12 607	(47.1)	6 580	(46.1)
Women	14 139	(52.9)	7 707	(53.9)
**Country, n (%)**				
Czech Republic	8 857	(33.1)	4 529	(31.7)
Poland	10 728	(40.1)	3 063	(21.4)
Lithuania	7 161	(26.8)	6 695	(46.9)
**Education, n (%)**				
Incomplete	129	(0.5)	28	(0.4)
Primary	3 111	(11.7)	908	(11.8)
Vocational	6 062	(22.8)	1 237	(16.1)
Secondary	9 081	(34.1)	2 597	(33.8)
College	1 731	(6.5)	1 054	(13.7)
University	6 490	(24.4)	1 866	(24.3)
**Occupational status, n (%)**				
Employed	8 031	(41.3)	5 768	(40.6)
Retired/employed	1 398	(7.2)	1 782	(12.6)
Retired/unemployed	9 227	(47.4)	5 982	(42.1)
Unemployed	800	(4.1)	669	(4.7)
**Smoking status, n (%)**				
Never	9 133	(50.6)	7 635	(53.7)
Past smoker	4 488	(24.9)	3 365	(23.7)
Current smoker	4 429	(24.5)	3 210	(22.6)
**Alcohol consumption[a], n (%)**				
Never	5 590	(31.2)	4 580	(32.5)
<1/monthly	4 509	(25.2)	3 644	(25.8)
1-3/monthly	3 653	(20.4)	2 923	(20.7)
1-4/weekly	3 109	(17.4)	2 246	(15.9)
≥5/weekly	1 051	(5.9)	720	(5.1)
Deprivation range[b], mean (SD)	1.5	2.4	1.4	2.2
**Physical activity[c], mean (SD)**				
Moderate	14.6	11.3	14.9	11.3
Vigorous	4.1	5.5	3.9	5.3
**Comorbidities, n (%)**				
Cardiovascular diseases				
Hypertension	11 676	(64.5)	9 121	(64.1)
Myocardial infarction	1 232	(6.9)	932	(6.6)
Ischemic heart disease	2 065	(11.6)	1 537	(10.9)
Stroke	607	(3.4)	483	(3.4)
Lung diseases				
COPD	2 558	(14.4)	2 025	(14.4)
Asthma	873	(4.9)	662	(4.7)
Other diseases				
Diabetes	1 837	(10.1)	1 327	(9.3)
Depression	17 569	(77.4)	9 233	(64.6)

COPD, chronic obstructive pulmonary disease.

[a] Alcohol consumption (never, graduated frequency from 1-3 drinks monthly or 1-5 drinks weekly).

[b]Deprivation scale (graded from 1 as a least deprived up to 12 as a most deprived).
[c]Number of hours of moderate and vigorous physical activity per week.
